# Exploring the Influences of Nurses’ Partnership with Parents, Attitude to Families’ Importance in Nursing Care, and Professional Self-Efficacy on Quality of Pediatric Nursing Care: A Path Model

**DOI:** 10.3390/ijerph17155452

**Published:** 2020-07-29

**Authors:** So Yeon Yoo, Haeryun Cho

**Affiliations:** 1 Department of Nursing, Kyungil University, Gyeongsan 38428, Korea; soyeon.yoo@gmail.com; 2 Department of Nursing, Wonkwang University, Iksan 54538, Korea

**Keywords:** nurse–parent partnership, attitude to families’ importance in nursing care, nursing professional self-efficacy, quality of pediatric nursing care, family-centered care

## Abstract

This study examined the effects of nurse–parent partnership, nurses’ attitude to families’ importance in nursing care, and nursing professional self-efficacy on the quality of pediatric nursing care. Background: The quality of pediatric nursing care based on family-centered care is defined by the qualitative care behavior of nurses from the perspective of hospitalized children and their families. Methods: The participants were 218 nurses in pediatric wards in hospitals. Data were collected using self-report questionnaires and analyzed using descriptive statistics, Pearson’s correlation coefficient, and path analysis. Results: Among the factors influencing the perceived quality of respect, explanation, and skillfulness, nurse experience showed the greatest total effect. The nurse–parent partnership had the greatest direct effect on the quality of respect and the greatest total effect on kindness. Nursing professional self-efficacy showed the greatest direct effect on explanation and the largest total effect on nurse–parent partnership and nurses’ attitude to families’ importance in nursing care. Conclusions: To improve the quality of pediatric nursing care, it is necessary to provide a working environment in which pediatric nurses can work continuously. Hospitals should also develop a program that enables proper collaboration between nurses and parents of hospitalized children and improves nursing professional self-efficacy.

## 1. Introduction

### 1.1. Background

Family-centered care (FCC) is decision-making for health care characterized by a partnership between the patient’s family and the health care professional, as well as respect and dignity for the patient’s cultural background, values, and beliefs [1]. Pediatric nursing care is based on the FCC philosophy. Therefore, when nursing hospitalized children, the nurse should respect and protect the dignity of the family and accurately assess nursing needs in accordance with the FCC philosophy [2,3]. In other words, nurses need to share all information without bias so that the family of hospitalized children can make a decision for nursing care and provide direct care in cooperation with the patient’s family; nurses must make decisions with and share the responsibilities of the patient’s family [3,4]. Parents of hospitalized children require nurses to respect the dignity of children and their parents and to provide an easy-to-understand explanation of treatment and nursing [5]. These parents also want to participate directly in the childcare process. These findings [1,2,3,4,5] provide the basis for nurses to apply the FCC in conducting high-quality childcare. 

Pediatric nurses must recognize the importance of the family and adopt a supportive attitude toward the family to practice FCC [6,7]. In Korea, where guardians staying in children’s wards is a common practice, the families of hospitalized children play an important role in the nursing care of children [5,8]. Pediatric nurses influence the quality of pediatric nursing care depending on their attitude toward the family of hospitalized children [9]. Therefore, the pediatric nurses’ attitude toward the importance of the family of children is a factor that can be considered when pediatric nurses want to improve the quality of pediatric nursing care based on FCC.

Indeed, the partnership between nurses and children’s parents is an important factor in the care and recovery of inpatient children. Partnership in this sense refers to the cooperative relationship between the pediatric nurse and the child’s parents that contributes to the provision of optimal nursing care for hospitalized children [10,11]. Providing care in collaboration with the parents of hospitalized children is an effective way to ensure the quality of nursing care provided for hospitalized children, regardless of residence of parents at the bedside [10,12]. A cooperative relationship between nurses and the parents of hospitalized children is an essential factor for managing the stress of children and families that is caused by hospitalization and providing qualitative nursing [8,13]. Indeed, the more positive the partnership between nurses and patient children’s parents, the higher the satisfaction with nursing service [10] and the quality of nursing care for hospitalized children [13]. Building positive partnerships between pediatric nurses and parents during hospitalization is closely related to the provision of the best FCC for inpatient children [11,14]. Therefore, the effects of the partnership between nurses and parents on the quality of pediatric nursing need to be confirmed.

Meanwhile, in the provision of quality nursing care, pediatric nurses’ professional self-efficacy has been considered an influencing factor. Self-efficacy is an individual’s belief in successfully being able to perform actions to achieve the desired outcome [15]. Professional self-efficacy refers to an individual’s confidence that he/she can perform qualitative outcomes for professional tasks assigned to the specific occupation [16,17]. Those with high professional self-efficacy successfully achieve the performance of the professional work of their job and have high job satisfaction [16]. In addition to the ability to handle the tasks required in special circumstances, these professionals also show a high level of quality in terms of decision-making [16,18]. Therefore, nurses with high self-efficacy can not only improve their professional nursing practical ability but also ultimately improve the quality of nursing care [17]. 

Research in pediatric nursing care based on FCC has attempted to develop concepts or guidelines for application to clinical settings [3,11,19,20,21]. Studies have also analyzed the perceptions of nurses, hospitalized children, and guardians of hospitalized children regarding the quality of pediatric nursing care based on FCC [5,13,14,22,23,24]. However, few studies have identified the factors that affect, directly and indirectly, the quality of pediatric nursing care to improve the quality of nursing care for hospitalized children.

Therefore, the present study aimed to identify the factors affecting the quality of pediatric nursing care based on FCC. Under this aim, we sought to investigate the effects of nurses’ attitude to families’ importance in nursing care, nursing professional self-efficacy, and nurse–parent partnership on the quality of nursing care for hospitalized children. The ultimate goal of this study was to prepare the basic data needed for developing strategies for improving the quality of nursing care for hospitalized children.

### 1.2. Hypothetical Model

**Figure 1:**
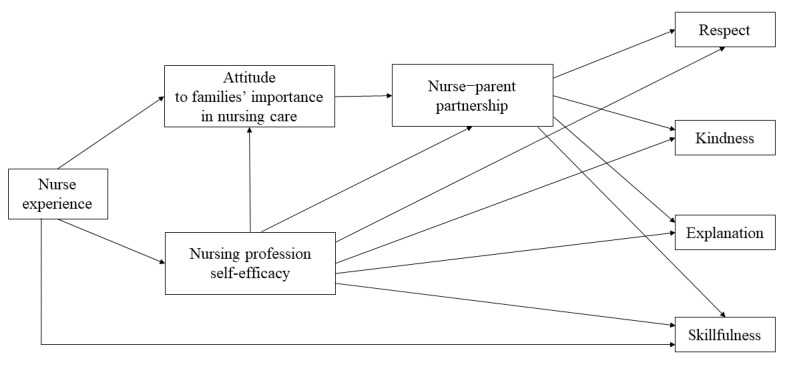
Hypothetical model of factors influencing quality of nursing care in the pediatric unit.

The hypothetical model of this study is presented in Figure 1. The research variables used in the hypothetical model were based on FCC conceptually: respect and dignity, participation, collaboration, and information sharing [1,25]. The quality of pediatric nursing care is a qualitative care performance of a nurse for the well-being of hospitalized children and their families [4,5]. Cho et al. [4], whose study was based on the FCC conceptual framework, reported that quality pediatric nursing care is characterized by skillful and kind nursing by a nurse who fully respects and provides easy explanation of procedures for hospitalized children and their families. The quality of pediatric nursing care, which was a dependent variable in the present study, refers to the respect, kindness, explanation, and skillfulness of the nurse. 

Respect, as referred to by FCC, is the acceptance and respect of the patient’s and family’s position and choices, which means planning and providing nursing care that considers the patient’s and family’s knowledge, values, beliefs, and cultural background [3,25]. In the context of quality of nursing care for inpatient children, respect means that nurses listen to and empathize with the inpatient children and their families, as well as provide nursing that matches the preference and needs of the children and caregivers based on a sense of trust [4].

In the same context of the quality of nursing care in pediatric wards, kindness is the expression of pediatric nurses: a kind and warm manner with interest in children and their families [4]. Pediatric nurses are required to not only provide sensitive responses to the physical and emotional needs of hospitalized children and their families but also adopt a careful attitude toward the latter for their well-being [10].

Meanwhile, explanation means that all nursing provided to hospitalized children, including the discharge plan and drug medication, is easy to understand [4]. A similar attribute of FCC is information sharing. FCC requires the indiscriminate sharing of the necessary information related to hospitalized children’s nursing care to inpatient children and their families [3,25].

Finally, skillfulness refers to the skilled nursing measures of pediatric nurses [4]. The quality of nursing care provided to hospitalized children is influenced by the nurses’ professional knowledge and skills [10].

Studies have confirmed that the nurse’s experience [14], nurse–parent partnership [8,13,14], attitudes to families’ importance in nursing care [3,5,20], and nursing professional self-efficacy [2,26,27] affect the quality of nursing in hospitalized children. Based on the literature review, a hypothetical model of this study was constructed.

## 2. Methods

### 2.1. Resarch Design

This study used a path analysis design to identify the factors that affect the quality of nursing care in the pediatric ward by applying patient- and family-centered nursing as the conceptual framework.

### 2.2. Research Participants

The participants of this study were nurses working in children’s wards at university hospitals and children’s specialty hospitals. Considering the hospitals distributed throughout the country, participants were sampled nurses working in the pediatric wards of three university hospitals in Seoul and Gyeonggi-do, two university hospitals in Gyeongsang-do, one university hospital and one children’s specialty hospital in Jeolla-do, and one university hospital in Gangwon-do. Bae [28] stated that at least 200 participants are required in path analysis with 12 or fewer observation variables. Our study used eight observational variables. We thus conducted a survey on 229 nurses in consideration of the dropout rate according to the criteria of Bae [28]. All the questionnaires distributed were collected; five questionnaires with insufficient responses were excluded, and six outliers with a standard score of ±2.0 or higher were eliminated in the review of the normality distribution of the data collected. Finally, data from 218 questionnaires were used for analysis, with the following distribution: 37.1% from Seoul and Gyeonggi, 29.4% from Gyeongsang, 27.5% from Jeolla, and 5% from Gangwon-do.

### 2.3. Research Instrument

#### 2.3.1. Attitude to Families’ Importance in Nursing Care

The instrument used in this study was the families’ importance in nursing care—pediatric nurses’ attitude scale that Oh et al. [6] validated in Korean. This instrument has 24 items and consists of six categories: family as a conversational partner, participant in care, supporter for the nurse, recipient of empowerment, burden, and resource. Each item is rated on a five-point Likert scale. Three items in family as a burden are calculated in reverse conversion. A higher score indicates that the nurse has a more positive attitude toward the family in pediatric nursing care. The Cronbach’s α for the reliability of the scale in Oh et al. [6] was 0.88, and in this study it was also 0.88.

#### 2.3.2. Nursing Professional Self-Efficacy 

The instrument used in this study was the Korean version of the nursing professions self-efficacy scale; validity and reliability were verified by translating the tools developed by Caruso et al. [17] to Korean. Italy, where the nursing professions self-efficacy scale was developed, and Korea have a similar nursing culture [17,29]. The English version of the nursing profession’s self-efficacy scale was translated to Korean using forward and backward translation. Subsequently, the content validity index (CVI) was assessed by six experts: two nursing professors, a nursing doctoral candidate, and three clinical nurses. The item CVI was from 0.71 to 1.0. The average item CVI was 0.98. The nursing professions self-efficacy scale has 19 items and two dimensions, namely, nursing situations and professional expertise situations. Each item is rated on a five-point Likert scale. Higher scores indicate higher nursing profession self-efficacy. The Cronbach’s ⍺ for reliability when the tool was developed was 0.83 [17], and it was 0.93 in this study.

#### 2.3.3. Nurse–Parent Partnership

Nurse–parent partnership was assessed using the pediatric nurse–parent partnership scale developed by Choi and Bang [10]. This scale consists of 34 items in seven categories, namely, cautiousness, shared information, communication, collaboration, sensitivity, professional knowledge and skill, and reciprocity. Each item is rated on a five-point Likert scale, with higher scores indicating a more positive partnership. The Cronbach’s α for the original scale was 0.92 [10], and the Cronbach’s ⍺ for the reliability shown in this study was 0.90.

#### 2.3.4. Quality of Nursing Care

The quality of nursing care was assessed using the instrument of quality of nursing care in pediatric wards modified by Yoo et al. [24] to target pediatric nurses. The original tool was developed by Cho et al. [4] to measure the quality of pediatric nursing care, targeted at parents of hospitalized children. The scale has 19 items in four categories, namely, respect, kindness, explanation, and skillfulness. The importance and performance of each items are measured on a four-point Likert scale. The score for the quality of nursing care is calculated as follows: 10 − (importance score × performance score). The total score ranges from 0 to 10, with higher scores indicating higher quality of nursing care. The Cronbach’s α for the reliability of the scale in Yoo et al. [24] was 0.94 for importance and 0.92 for performance. In the present study, Cronbach’s α was 0.92 for importance and 0.91 for performance.

### 2.4. Data Collection and Ethical Considerations

After obtaining approval from the Institutional Review Board of W University (IRB no. WKIRB- 201806-SB-044), this study was conducted between 30 July and 24 August 2018. The purpose of the study was explained to the heads of the institutions of the seven university hospitals and one children’s specialty hospital. The researchers visited the wards for the data collection. The pediatric nurses were provided with a verbal explanation of the purpose of the study, their rights regarding consent and refusal with respect to their voluntary participation in the study, and the possibility of abandonment, and so on. Those who voluntarily agreed to participate in the study were asked to fill out a written consent form and then to complete a questionnaire, which took about 10 min. After completing the questionnaire, each respondent was given approximately 8 USD worth of goods as a token of appreciation.

### 2.5. Data Analysis

The collected data were analyzed using IBM SPSS Statistics 24 (IBM Crop., Armonk, NY, USA) and IBM SPSS AMOS 24 (IBM Crop., Armonk, NY, USA). Descriptive statistics for the general characteristics of the participants, endogenous variables, and exogenous variables were presented as frequency, percentage, mean, and standard deviation values. The normality review of the study variables was calculated by checking the standard score, skewness, and kurtosis. The correlation and multicollinearity among each variable were confirmed using Pearson’s correlation coefficient. In addition, multicollinearity was confirmed using tolerance limits and variation inflation factor (VIF) values. To confirm the goodness of fit of the hypothetical model and modified model of this study, we identified the absolute fit indexes χ^2^ and χ^2^/df, goodness-of-fit index (GFI), adjusted goodness-of-fit index (AGFI), root mean squared error of approximation (RMSEA), comparative-fit index (CFI), normed-fit index (NFI), and Tucker–Lewis index (TLI). The path coefficient estimation and effect analysis were performed using the maximum-likelihood method. The bootstrapping method was used to verify the significance of the direct and indirect effects and the total effect.

## 3. Results

### 3.1. General Characteristics of Participants

The mean age of the nurses in the pediatric wards that participated in this study was 29.20 ± 7.07 years; 72.9% were unmarried and 82.6% had no children. Among the participants, 83.5% were general nurses, 11.9% were charge nurses, and 4.6% were head nurses. The average experience in the pediatric ward was 47.06 ± 56.46 months, and the total experience of nurses was 79.94 ± 84.02 months (Table 1).

### 3.2. Descriptive Statistics of Study Variables

The average scores for attitude to families’ importance in nursing care, nursing professional self-efficacy, nurse–parent partnership were 85.81 ± 9.62, 71.42 ± 8.53, and 130.95 ± 13.55, respectively. The average score for quality of nursing in pediatric units was 8.14 ± 0.77; by category, respect was scored 8.11 ± 0.91; kindness, 8.30 ± 1.10; explanation, 8.28 ± 0.95; and skillfulness, 7.69 ± 1.19 (Table 1). The skewness of all variables was within ±1.965, and the kurtosis was close to 0, indicating that the data met the criteria for normal distribution [30].

### 3.3. Correlation of Study Variables

Table 2 shows the correlation between the research variables. There was a significant correlation between all variables except for the total experience of nurses, quality of kindness, attitude to families’ mportance in nursing care, and quality of explanation. When examining the correlation coefficient to determine multicollinearity, we found that the largest correlation coefficient was 0.69, which was the correlation coefficient between nursing professional self-efficacy and nurse–parent partnership, and it stayed below 0.70. In addition, the tolerance limit exceeded 0.1 with 0.47–0.85, and VIF was 1.18–2.14, which was less than 10, indicating the absence of multicollinearity [28].

### 3.4. Fitness of Hypothetical Model

The model fitness of the hypothetical model of this study was indicated as follows: χ^2^ = 23.02 (*p* = 0.003), χ^2^/df = 2.88, GFI = 0.98, AGFI = 0.90, CFI = 0.97, NFI = 0.96, TLI = 0.90, RMSEA = 0.09. Among the fit indexes, GFI, AFGI, CFI, NFI, and TLI were good levels, exceeding 0.90, whereas RMSEA was moderate. Meanwhile, the significance probability of χ^2^ was less than 0.05, and χ^2^/df exceeded 2.0, requiring model modification. In addition, five of the total 14 paths were not statistically significant. Thus, the model was modified.

### 3.5. Model Modification 

**Figure 2:**
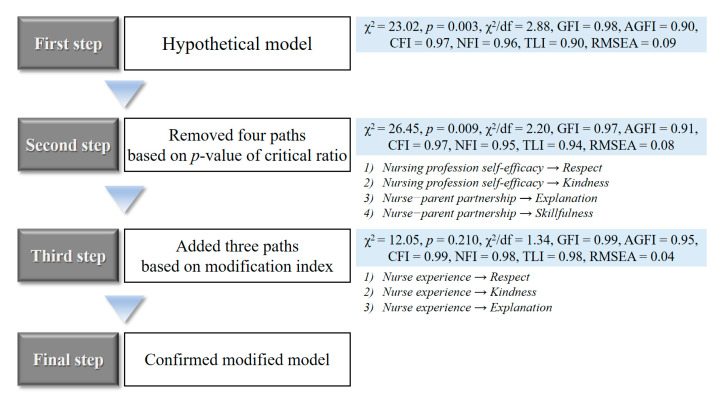
Flow of modification in this study.

The model modification process is shown in Figure 2. The existing variables were maintained and modified stepwise using a critical ratio and modification index, in consideration of the logical validity and theoretical background of the model. For simplicity, the optimal model was identified by modifying each path.

First, using a fixed index, the path between the nursing professional self-efficacy and respect (i.e., of quality of nursing care), which was not statistically significant, and that between kindness and nursing professional self-efficacy were deleted. The path between nurse–parent partnership and explanation and that between nurse–parent partnership and skillfulness were deleted as well. The path between the total nurse experience and attitude to families’ importance in nursing care was not statistically significant. However, after literature review and in-depth discussion between the researchers, we decided that nurses’ attitude to families’ importance in nursing care should not be deleted owing to its importance in the performance of patient- and family-centered care. At this stage, four paths were deleted, and the model fitness showed a good level of RMSEA. However, the significance probability of χ^2^ was still less than 0.05, and χ^2^/df also exceeded 2.0. Thus, further modification was needed.

Second, we added the path between nurse experience and respect, that between nurse experience and kindness, and that between nurse experience and explanation by referring to logical validity, theoretical background, and modification index. Although the path between nurse experience and kindness was not statistically significant, it was decided not to delete it from the model after comparing the logical validity and nurse experience and the contents of the questions measuring kindness. At this stage, three paths were added, the RMSEA level improved, the significance probability of χ^2^ exceeded 0.05, and χ^2^/df was less than 2.0.

### 3.6. Fitness of Modified Model

**Figure 3:**
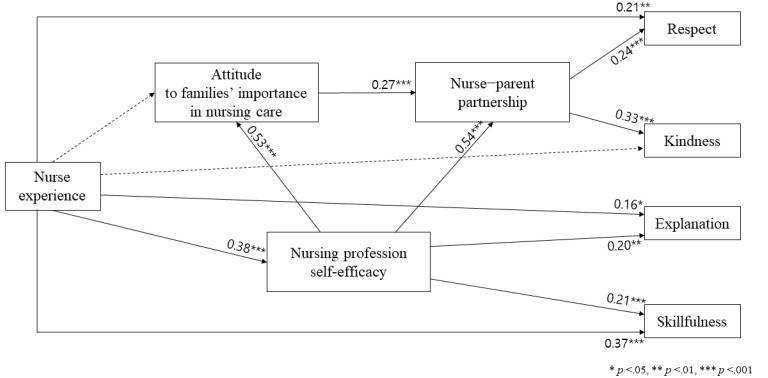
Modified model of factors influencing quality of nursing care in pediatric units.

The final model was modified within the range of the theoretical basis, logical validity, and model simplicity by referring to the GFI, statistical significance of the critical ratio, and modification index (Figure 3). The goodness of fit of the modified model was shown as follows: χ^2^ = 12.05 (*p* = 0.210), χ^2^/df = 1.34, GFI = 0.99, AGFI = 0.95, CFI = 0.99, NFI = 0.98, TLI = 0.98, RMSEA = 0.04. Thus, the criteria of goodness of fit were as follows: *p*-value of χ^2^ of 0.05 or more, χ^2^/df of 2 or less, and GFI, AGFI, CFI, NFI, and TLI of 0.09 or more, and RMSEA of 0.05 or less. When the modification indices of the modified model were confirmed, no further paths could be added.

### 3.7. Path Coefficient and Effect of Modified Model 

Figure 3 shows the path coefficient of the path model for factors affecting the quality of nursing care in pediatric units. The direct, indirect, and total effects of the research variables are presented in Table 3. Regarding the total effect on respect, all factors had a significant effect: nurse experience (β = 0.28, *p* = 0.004), nurse–parent partnership (β = 0.24, *p* = 0.004), nursing professional self-efficacy (β = 0.17, *p* = 0.004), and attitude to families’ importance in nursing care (β = 0.07, *p* = 0.004); the explanatory power was 13%. Nurse experience showed significant indirect effects on kindness (β = 0.09, *p* = 0.004) but the total effect was not significant (β = 0.10, *p* = 0.129). Meanwhile, nurse–parent partnership (β = 0.33, *p* = 0.004), nursing professional self-efficacy (β = 0.22, *p* = 0.004), and attitude to families’ importance in nursing care exerted an influence on kindness, and the explanatory power was 11%. The explanation was influenced by the nurse experience (β = 0.24, *p* = 0.004) and nursing professional self-efficacy (β = 0.20, *p* = 0.014), in order, and the explanatory power was 9%. Finally, skillfulness had a significant influence: nurse experience (β = 0.45, *p* = 0.004) and nursing professional self-efficacy (β = 0.21, *p* = 0.004); the explanatory power was 25%.

Looking at the total effect on the mediating variables, the factors that significantly influenced the nurse–parent partnership were nursing professional self-efficacy (β = 0.68, *p* = 0.004), nurse experience (β = 0.28, *p* = 0.004), attitude to families’ importance in nursing care (β = 0.27, *p* = 0.004), and the explanatory power was 52%. The attitude to families’ importance in nursing care was the most influenced by nursing professional self-efficacy (β = 0.53, *p* = 0.004), whereas nurse experience was not significant (β = 0.07, *p* = 0.208) but had a significant total effect (β = 0.20, *p* = 0.004) and showed an explanatory power of 31%. Finally, nursing professional self-efficacy was significantly influenced by nurse experience (β = 0.38, *p* = 0.004), and the explanatory power was 14%.

## 4. Discussion

The present study aimed to identify the path model for examining the factors that affect the quality of nursing care for hospitalized children based on FCC. The goal of this study was to provide the basic data needed to develop strategies for improving the quality of nursing care for inpatient children. Respect, among the subtypes of the quality of nursing care in pediatric units for hospitalized children, was found to be influenced by the following, respectively: nurse experience, nurse–parent partnership, nursing professional self-efficacy, and attitude to families’ importance in nursing care. Kindness, another subtype, was influenced by the following, respectively: nurse–parent partnership, nursing professional self-efficacy, and attitude to families’ importance in nursing care; the experience of the nurse had no significant effect. Explanation, another of the subtypes of quality of nursing care, was influenced by the following, respectively: nurse experience and nursing professional self-efficacy. As for the subtype skillfulness, it was influenced by the following, respectively: nurse experience and nursing professional self-efficacy. 

Nurses’ experience, in other words, nurses’ careers, showed the greatest total effect on the quality of respect, explanation, and skillfulness. This result coincides with that of Yoo et al. [14], which reported that the quality of nursing care is high for experienced nurses. The characteristics of nurses are reported to be closely related to the quality of nursing provided to hospitalized children [22]. Indeed, senior nurses with much professional experience in pediatric nursing are well-adjusted to FCC through personal experience with marriage, parenting, or academic endeavor, and they have been shown to build better partnerships with inpatient children’s parents [8,27]. As nurses progress through their career, they experience a variety of patient and nursing situations. This experience not only makes nurses more understanding of human dignity and respect for patients and their families but also enhances their ability to perform more skilled nursing activities, thereby improving the quality of nursing care [14]. Meanwhile, new nurses have short nursing experience, and as such, they are unfamiliar with many nursing circumstances and patients, and they may be uneasy with what would happen in the future, as well as tend to have difficulty performing qualitative nursing [31]. Therefore, a strong strategy would be to place nurses who have sufficient nursing experience in the pediatric ward to provide qualitative pediatric nursing care. However, in reality, if such personnel arrangement is difficult, additional education may be provided to strengthen the skills required for nurses to care for hospitalized children and the basic nursing capacity for pediatric nurses with short experience. 

Regarding the quality of nursing care for hospitalized children, nurse–parent partnerships had the greatest direct effect on respect and the greatest total effect on kindness. This result was similar to that in a study on a mother–nurse partnership program for children, and their mothers, admitted to the intensive care unit for congenital heart disease [12]. The program was reported as effective in terms of communication, mutual dependence, cooperation, information sharing, and careful and sensitive consideration [12]. The finding of this study was also similar to the results of Yoo et al. [14], which reported that the higher the partnership between parents and nurses, the higher the quality of nursing care for hospitalized children. The result of this study was that nurse–parent partnership affected the quality of respect and kindness, among the four categories of quality of nursing care in pediatric units, in turn reflecting the fact that inpatient nursing ought to be based on FCC. In Korea, parents stay with their children during hospitalization to reduce the separation anxiety and ensure the physical and psychological stability of inpatient children during hospitalization [5,8]. Participation in nursing care, in collaboration with nurses, by families residing near inpatient children improves not only the satisfaction with nursing care of the children and families but also the quality of pediatric nursing care [20]. Pediatric nurses establishing meaningful relationships with inpatient children’s parents is, thus, a key element for quality nursing care [2]. Therefore, nurses and parents must build a positive partnership for the qualitative nursing of inpatient children.

Nursing professional self-efficacy had the greatest direct effect on explanation, and the largest total effect on attitude to families’ importance in nursing care and nurse–parent partnership. Cheng et al. [32] reported that the higher the self-efficacy perceived by pediatric nurses, the higher the professional perception, and the better the relationship between nurses and patients. Alavie et al. [26], in their analysis of the concept of pediatric nurses’ care self-efficacy, highlighted the importance of the provision of sufficient explanations to hospitalized children and their families, which improves the self-efficacy of child care to such a degree that FCC is fully implemented. Based on these previous findings, the result in this study could be explained as follows: nursing professional self-efficacy in pediatric nurses affected their attitude toward the importance of the patient’s family and nurse–parent partnership. In addition, professional communication is considered one of the main attributes of the self-efficacy of pediatric nurses [2]. Parents of inpatient children have a high demand for detailed explanations of everything related to their child’s care [5,24]. Therefore, the quality of inpatient childcare will be improved by improving the nursing professional self-efficacy of pediatric nurses. 

In this study, the data were collected from nurses belonging to hospitals located across the country. Thus, the strength of the present research is the representativeness of the data. In addition, we confirmed how the concepts of FCC had an effect on the quality of actual child nursing care by setting variables based on FCC, the philosophical foundation of child nursing care. However, the squared multiple correlations, or the explanatory power of the model verified in this study, was slightly low. This limitation can be interpreted as reflecting the reality that FCC remains abstract in the actual clinical field [19,21,23,24]. In this regard, continuous education and empirical research should be conducted until FCC takes root in the clinical field. In addition to the variables considered in this study, future research is necessary to develop a model with a sufficient level of explanatory power for exploring and considering the various factors that can affect the quality of nursing care in pediatric units. Finally, the concept in this study considered only the parent. Because the concept of children’s parents is expanding to include guardians, guardians of hospitalized children need to be considered in a future study. 

## 5. Conclusions

This study was conducted to identify the factors influencing the quality of nursing care in pediatric units and to provide fundamental data necessary for developing strategies for improving the quality of nursing for hospitalized children. Based on the main results, the implications for improving the quality of nursing care for hospitalized children were as follows. First, nurse experience was a major influencing factor of the quality of pediatric nursing care. Therefore, hospitals should prepare a working environment in which pediatric nurses could work for a long time. Second, for junior pediatric nurses, hospitals should develop a program that can build better partnerships between nurses and parents of hospitalized children and improve the self-efficacy of nursing professionals in providing better pediatric care. Through such a program, improving the competence for building nurse–parent partnership and self-efficacy of pediatric nurses will be a strategy, in particular, for junior pediatric nurses to compensate for their lack of experience. Therefore, junior nurses with insufficient nursing experience will be able to provide quality pediatric nursing care in the ward. 

## Figures and Tables

**Table 1:** General characteristics of pediatric nurses and descriptive statistics of the measured variables (*N* = 218).

Categories	n (%)/M ± SD	Min–Max	Range	Skewness	Kurtosis
General Characteristics					
Age (years)	29.20 ± 7.07				
Marital status					
Single	159 (72.9)				
Married	59 (27.1)				
Child					
None	180 (82.6)				
At least one	38 (17.4)				
Education					
College	57 (26.1)				
Undergraduate	149 (68.3)				
Graduate	12 (5.5)				
Position					
Staff nurse	182 (83.5)				
Charge nurse	26 (11.9)				
Head nurse	10 (4.6)				
Pediatric experience (months)	47.06 ± 53.46				
Nurse experience (months)	79.94 ± 84.02				
Measured Variables					
Attitude to families’ importance in nursing care	85.81 ± 9.62	57.00–111.00	54.00	−0.10	0.22
Nursing professional self-efficacy	71.42 ± 8.53	46.00–95.00	49.00	0.23	0.38
Nurse–parent partnership	130.95 ± 13.55	95.00–168.00	73.00	−0.17	0.22
Quality of nursing care	8.14 ± 0.77	6.25–10.00	3.75	0.15	−0.16
Respect	8.11 ± 0.91	5.78–10.00	4.22	0.23	−0.11
Kindness	8.30 ± 1.10	5.09–10.00	4.91	−0.12	−0.41
Explanation	8.28 ± 0.95	5.98–10.00	4.02	−0.21	−0.64
Skillfulness	7.69 ± 1.19	3.99–10.00	6.01	−0.37	0.17

**Table 2:** Correlation, tolerance, and variation inflation factor among the measured variables (*N* = 218).

Variables	Nurse Experience	Attitude to Families’ Importance in Nursing Care	Nursing Professional Self-Efficacy	Nurse–Parent Partnership
	r(*p*)	r(*p*)	r(*p*)	r(*p*)
Attitude to families’ importance in nursing care	0.27 (<0.001)			
Nursing professional self-efficacy	0.38 (<0.001)	0.56 (<0.001)		
Nurse–parent partnership	0.32 (<0.001)	0.57 (<0.001)	0.69 (<0.001)	
Quality of nursing care				
Respect	0.29 (<0.001)	0.29 (<0.001)	0.24 (<0.001)	0.32 (<0.001)
Kindness	0.11 (0.099)	0.20 (0.004)	0.31 (<0.001)	0.35 (<0.001)
Explanation	0.24 (<0.001)	0.13 (0.053)	0.29 (<0.001)	0.23 (0.001)
Skillfulness	0.45 (<0.001)	0.19 (0.004)	0.37 (<0.001)	0.27 (<0.001)
Tolerance	0.85	0.62	0.47	0.48
Variation inflation factor	1.18	1.60	2.14	2.09

r = correlation coefficient, *p* = probability value

**Table 3:** Estimates and standardized effect of the modified model.

Endogenous Variables	Standardized Estimate	Standard Error	Critical Ratio (*p*)	Squared Multiple Correlations	Direct Effect (*p*)	Indirect Effect (*p*)	Total Effect (*p*)
Exogenous Variables							
Attitude to families’ importance in nursing care				0.31			
Nurse experience	0.07	0.007	1.21 (0.226)		0.07 (0.208)	0.20 (0.004)	0.27 (0.004)
Nursing professional self-efficacy	0.53	0.069	8.67 (<0.001)		0.53 (0.004)	-	0.53 (0.004)
Nursing professional self-efficacy				0.14			
Nurse experience	0.38	0.006	6.02 (<0.001)		0.38 (0.004)	-	0.38 (0.004)
Nurse–parent partnership				0.52			
Nurse experience	-	0.001	-		-	0.28 (0.004)	0.28 (0.004)
Attitude to families’ importance in nursing care	0.27	0.080	4.78 (<0.001)		0.27 (0.004)	-	0.27 (0.004)
Nursing professional self-efficacy	0.54	0.090	9.46 (<0.001)		0.54 (0.004)	0.14 (0.004)	0.68 (0.004)
Quality of nursing care in pediatric unit							
Respect				0.13			
Nurse experience	0.21	0.001	3.21 (0.001)		0.21 (0.004)	0.07 (0.004)	0.28 (0.004)
Attitude to families’ importance in nursing care	-	-	-		-	0.07 (0.004)	0.07 (0.004)
Nursing professional self-efficacy	-	-	-		-	0.17 (0.004)	0.17 (0.004)
Nurse–parent partnership	0.24	0.004	3.95 (<0.001)		0.24 (0.004)	-	0.24 (0.004)
Kindness				0.11			
Nurse experience	0.01	0.001	0.13 (0.897)		0.01 (0.885)	0.09 (0.004)	0.10 (0.129)
Attitude to families’ importance in nursing care	-	-	-		-	0.09 (0.004)	0.09 (0.004)
Nursing professional self-efficacy	-	-	-		-	0.22 (0.004)	0.22 (0.004)
Nurse–parent partnership	0.33	0.005	5.35 (<0.001)		0.33 (0.004)	-	0.33 (0.004)
Explanation				0.09			
Nurse experience	0.16	0.001	2.34 (0.019)		0.16 (0.022)	0.08 (0.014)	0.24 (0.004)
Nursing professional self-efficacy	0.20	0.007	3.14 (0.002)		0.20 (0.014)	-	0.20 (0.014)
Skillfulness				0.25			
Nurse experience	0.37	0.001	5.92 (<0.001)		0.37 (0.004)	0.08 (0.004)	0.45 (0.004)
Nursing professional self-efficacy	0.21	0.008	3.52 (<0.001)		0.21 (0.004)	-	0.21 (0.004)

*p* = probability value

## References

[1] Kuo D.Z., Houtrow A.J., Arango P., Kuhlthau K.A., Simmons J.M., Neff J.M. (2012). Family-centered care: Current applications and future directions in pediatric health care. Matern. Child Health J..

[2] Alivi A., Bahrami M., Boroujeni Z.A., Yousefy A. (2015). Characteristics of caring self-efficacy in pediatric nurses: A qualitative study. J. Spec. Pediatr. Nurs..

[3] Hill C., Knafl K.A., Santacroce S.J. (2018). Family-centered care from the perspective of parents of children cared for in a pediatric intensive care unit: An integrative review. J. Pediatr. Nurs..

[4] Cho H., Oh J., Jung D. (2015). Development of an instrument to measure the quality of care through patients’ eyes for hospitalized child. Child Health Nurs. Res..

[5] Kim Y.Y., Cho H. (2017). A convergence study on nursing needs of hospitalized children’s mothers and quality of care in pediatric unit. J. Korea Converg. Soc..

[6] Oh J., Kim Y.Y., Yoo S.Y., Cho H. (2018). Validity and reliability of the Korean version of the families’ importance in nursing care-pediatric nurses’ attitudes instrument. Child. Health Nurs. Res..

[7] Saveman B.I., Benzein E.G., Engström A.H., Arestedt K. (2011). Refinement and psychometric reevaluation of the instrument: Families’ importance in nursing care-nurses’ attitudes. J. Fam. Nurs..

[8] Choi M., Kim J. (2014). Associated factors in pediatric nurse parent partnership. Child. Health Nurs. Res..

[9] Coyne I., O’Neill C., Murphy M., Costello T., O’shea R. (2011). What does family-centered care mean to nurses and how do they think it could be enhanced in practice. J. Adv. Nurs..

[10] Choi M.Y., Bang K.S. (2013). Development and testing of a pediatric nurse parent partnership scale. J. Korean Acad. Nurs..

[11] Tallon M.M., Kendall G.E., Sinder P.D. (2015). Rethinking family-centered care for the child and family in hospital. J. Clin. Nurs..

[12] Uhm J., Kim H.S. (2019). Impact of the mother-nurse partnership programme on mother and infant outcomes in paediatric cardiac intensive care unit. Intensiv. Crit. Care Nurs..

[13] Bae S.Y., Lee I. (2017). The effect of child’s mother and nurse partnership on the anxiety and perceived quality of nursing care of hospitalized child’s mother. J. Korea Acad. Ind. Coop. Soc..

[14] Yoo S.Y., Cho H., Kim Y.Y., Park J.H. (2020). Levels of partnership between nurses and parents of hospitalized children and the quality of pediatric nursing care as perceived by nurses. Child Health Nurs. Res..

[15] Bandura A., Pajares F., Urdan T.S. (2006). Guide for constructing self-efficacy scales. Self-Efficacy Beliefs of Adolescents.

[16] Bargsted M., Vielma R.R., Yeves J. (2019). Professional self-efficacy and job satisfaction: The mediator role of work design. J. Work Organ. Psy..

[17] Caruso R., Pittella F., Zaghini F., Fida R., Sili A. (2016). Development and validation of the nursing profession self-efficacy scale. Int. Nurs. Rev..

[18] Lu M., Zou Y., Chen X., Chen J., He W., Pang F. (2020). Knowledge, attitude and professional self-efficacy of chinese mainstream primary school teachers regarding children with autism spectrum disorder. Res. Autism Spectr. Disord..

[19] Boztepe H., Kerimoğlu Y.G. (2017). Nurses’ perceptions of barriers to implementing family-centered care in a pediatric setting: A qualitative study. J. Spec. Pediatr. Nurs..

[20] Davidson J.E., Aslakson R.A., Long A.C., Puntillo K.A., Kross E.K., Hart J., Cox C.E. (2017). Guidelines for family-centered care in the neonatal, pediatric, and adult ICU. Crit. Care Med..

[21] Smith W. (2018). Concept analysis of family-centered care of hospitalized pediatric patients. J. Pediatr. Nurs..

[22] Comparcini D., Simonetti V., Tomietto M., Kilpi L.H., Pelander T., Cicolini G. (2018). Children’s perceptions about the quality of pediatric nursing care: A large multicenter cross-sectional study. J. Nurs. Sch..

[23] Dall’Oglio I., Furia M., Tiozzo E., Gawronski O., Biagiolo V., Ciommo D.V.M., Paoletti S., Bianchi N., Celesti L., Raponi M. (2018). Practice and perceptions of family centered care among healthcare providers: A cross-sectional study in a pediatric hospital. J. Pediatr. Nurs..

[24] Yoo S.Y., Kim Y.Y., Cho H. (2018). Comparison of the quality of nursing care as perceived by pediatric nurses and mothers of hospitalized children. Child. Health Nurs. Res..

[25] Johnson B., Abraham M., Conway J., Simmons L., Levitan E.S., Sodomka P. (2008). Partnering with Patients and Families to Design a Patient- and Family-Centered Health Care System: Recommendations and Promising Practices.

[26] Alavi A., Boroujeni Z.A., Yousefy A., Bahrami M. (2017). Altruism, the values dimension of caring self-efficacy concept in Iranian pediatric nurses. J. Educ. Health Promot..

[27] Hong E., Yang Y. (2015). Factors affecting job stress of pediatric nurses: Focusing on self-efficacy, emotional labor, pediatric nurse-partnership. Child. Health Nurs. Res..

[28] Bae B.R. (2017). Structural Equation Modeling with Amos 24.

[29] Cho S., Lee J., June K., Hong K.J., Kim Y. (2016). Nurse staffing levels and proportion of hospitals and clinics meeting the legal standard for nurse staffing for 1996–2013. J. Korean Acad. Nurs. Adm..

[30] Han S.S., Lee S.C. (2012). Nursing and Health Statistical Analysis.

[31] Lee H., Kim S.J., Park S.Y. (2017). Newly nurses’ experience in their first year of practice. J. Qual. Res..

[32] Cheng L., Cui Y., Chen Q., Ye Y., Liu Y., Zhang F. (2020). Paediatric nurses’ general self-efficacy. perceived organizational support and perceived professional benefits from class a tertiary hospitals in Jilin province of China: The mediating effect of nursing practice environment. BMC Health Serv. Res..

